# Searching for Principles of Microbial Ecology Across Levels of Biological Organization

**DOI:** 10.1093/icb/icad060

**Published:** 2023-06-06

**Authors:** Matti Gralka

**Affiliations:** Systems Biology lab, Amsterdam Institute for Life and Environment (A-LIFE), Amsterdam Institute of Molecular and Life Sciences (AIMMS), Vrije Universiteit Amsterdam, 1081 HV, The Netherlands

## Abstract

Microbial communities play pivotal roles in ecosystems across different scales, from global elemental cycles to household food fermentations. These complex assemblies comprise hundreds or thousands of microbial species whose abundances vary over time and space. Unraveling the principles that guide their dynamics at different levels of biological organization, from individual species, their interactions, to complex microbial communities, is a major challenge. To what extent are these different levels of organization governed by separate principles, and how can we connect these levels to develop predictive models for the dynamics and function of microbial communities? Here, we will discuss recent advances that point towards principles of microbial communities, rooted in various disciplines from physics, biochemistry, and dynamical systems. By considering the marine carbon cycle as a concrete example, we demonstrate how the integration of levels of biological organization can offer deeper insights into the impact of increasing temperatures, such as those associated with climate change, on ecosystem-scale processes. We argue that by focusing on principles that transcend specific microbiomes, we can pave the way for a comprehensive understanding of microbial community dynamics and the development of predictive models for diverse ecosystems.

## Main text

Microbial communities are ubiquitous across all ecosystems on the planet and play crucial roles in various ecosystem functions. They cycle elements such as carbon and nitrogen in ecosystems from the oceans to soils (Falkowski et al. 2008); they impact animal and plant health, and they are important for industrial processes, from food (e.g., fermentations) to biofuels. Understanding microbial communities at a deeper level is essential for predicting their response to environmental changes (e.g., climate change), to design interventions to improve human health, and constructing communities from scratch for specific purposes like enhancing the flavor of plant-based meats.

Modern sequencing capabilities and extensive sampling efforts, including dedicated ocean cruises (Sunagawa et al. 2015) and large-scale collaborations like the Earth Microbiome Project (Thompson et al. 2017) and the Human Microbiome Project (Turnbaugh et al. 2007; Proctor et al. 2019), have provided unprecedented insight into the composition of microbial communities. However, community compositions obtained by metagenome or amplicon sequencing only provide relative abundance data, offering little information about the traits and activity of the observed organisms, their interactions, and how their collective actions drive the function of a given community. To address this limitation, we propose a synergistic approach of investigating communities at three different levels of organization: individual traits, interactions, and complex communities.

In the following, we will discuss recent advances in our understanding of microbial communities from a perspective of community metabolism. We will argue that efforts must be directed towards discovering *principles*, concepts that are rooted in physics or biochemistry and therefore transcend specific microbiomes. These principles may be specific to each level of organization, and we will therefore go on a journey through the scales, from individual populations to whole communities.

### Individual species

To establish a comprehensive theory of microbial communities from the bottom up, it is natural to begin by examining the traits of individual species. These traits include a wide range of characteristics, including growth rates under various conditions, biofilm formation, motility, antibiotic resistance, nitrogen fixation, and many others. The specific traits of interest depend on the microbiome under study. Here, we focus on the metabolism and physiology of bacteria and other heterotrophic microbes. We choose this focus because metabolic interactions are at the heart of microbial communities, and metabolic biochemistry follows universal principles, such that starting with knowledge about microbial metabolism provides a foundation for understanding microbial communities.

A key goal of microbial physiology is to explain the differences and commonalities between organisms in terms of fundamental principles. Phenomenological models capture physiological processes in terms of coarse-grained features of the cells, such as the relative allocation of different kinds of enzymes and their quantitative effects on the molecular composition in terms of protein, RNA, and DNA, or the rate of switching between substrates (Scott et al. 2014; Basan et al. 2015; Basan 2018; Basan et al. 2020). These models are based on a few simple principles, such as a fixed total amount of enzymes per cell and a cellular objective of maximizing growth rate. These principles emerge from quantitative experiments that carefully control all environmental variables, and appear to be universal across many microbes tested (Bruggeman et al. 2020).

However, even for well-studied microbes, many detailed observations, such as the abundances of individual metabolic enzymes as a function of growth conditions, remain unexplained (Mori et al. 2021). Complexity increases further when conditions deviate from balanced growth, a carefully controlled state where all biological processes are in steady-state. Gene expression regulation, which is often not fully understood (even in *E. coli*, the arguably best understood organism on the planet, Belliveau et al. 2021), leads to diverse cellular behaviors under different environmental conditions and transient behaviors upon environmental change, possibly varying among clonal members of the same population.

Furthermore, microbes in the wild differ substantially from *E. coli* and each other in various ways, making it unclear which principles learned from *E. coli* can be directly applied to other, potentially distantly related microbes. Two primary ways provide insight into the metabolic capabilities and physiology of non-model microbes: direct phenotypic characterization (largely restricted to culturable microbes), and inference from genomic data.

Direct phenotypic characterization of diverse microbes has led to the development of large databases of microbial traits based on experimental characterization (Barberán et al. 2017; Madin et al. 2020; Heinken et al. 2023). However, these databases suffer from several issues. They lack completeness, as not every trait is measured for every species. Further, traits are not always measured using standardized methods. For instance, trait databases may contain information on suitable growth substrates for a given species, but information on which substrates were tested is often unavailable. Finally, quantitative data, such as growth rates and their dependence on environmental parameters, is rarely available. Therefore, experimental characterization of non-model organisms remains an important pillar of microbial ecology.

Experimental characterization of wild microbes can uncover underlying principles guiding the evolution of metabolic phenotypes. For example, a recent study characterizing the growth capabilities of 186 diverse marine heterotrophic bacteria on 140 carbon sources (Gralka et al. 2022) revealed a candidate principle in the context of carbon catabolism: the carbon catabolic strategies of heterotrophic bacteria can be summarized in terms of their preferences for either sugars or acids. This preference arises from distinct metabolic enzymes involved in glycolysis and gluconeogenesis and the conflicting nature of these activities in the cell (Basan et al. 2020; Schink et al. 2022). By annotating community compositions in terms of the metabolic preferences of their constituents, hypotheses can be generated about potential interactions and the dominant carbon catabolic processes within a given community. Thus, coarse-grained metabolic traits rooted in the principles of microbial physiology may enable insight into the metabolic processes inside communities.

In parallel to experimental characterization, certain traits can be inferred from genomic information. These genotype-to-phenotype mappings build upon experimental characterizations and enable trait predictions for species that have not (yet) been cultured. Such predictions enable the functional annotation of communities using metagenomic datasets. Metabolic differences between well-studied organisms are captured in genome-scale metabolic models (GEMs) by assembling all known metabolic reactions into a model. GEMs provide deep insight into the physiology of individual species, including gene essentiality, metabolic fluxes, or predicted excretions. Consequently, GEMs are a vital tool in both basic and applied microbiology. However, while initial model construction is now mostly automated, accurate models require extensive curation (Machado et al. 2018; Heirendt et al. 2019; Bekiaris and Klamt 2020; Karp et al. 2021; Seaver et al. 2021), making them generally unavailable for a given species. However, by incorporating available phenotype data and knowledge from closely related species, curated genome-scale metabolic models have now been developed for numerous bacteria (Poyet et al. 2019; Heinken et al. 2023).

At present, it remains very challenging to accurately estimate a given species’ metabolic and behavioral repertoire from its genome alone. This difficulty stems at least in part from the fact that small genotypic changes can give rise to large phenotypic changes. For example, in the above-mentioned experimental characterization of 186 bacteria, individual growth capabilities were not predictable, but the preference for glycolytic versus gluconeogenic substrates could be predicted from genome content, even for organisms not directly characterized. This highlights a recurring theme: while certain details (e.g., exact phenotypes from genomes, but also abundances of species, see below) may be practically or even fundamentally impossible to predict, predictability can be restored by focusing on appropriately coarse-grained descriptors of individual or community characteristics, such as general metabolic preferences or the abundance of certain functions in the community.

### Bottom-up view of interactions and communities

Interactions between microbes are foundational to their communities. Therefore, it is important to find effective ways of understanding the principles that underlie them, inferring them from genomic data, and predicting how they change with the environment. Models of species interactions can be broadly grouped into two categories: models with fixed, direct interactions (e.g., Lotka-Volterra model), and models with explicitly resource-mediated interactions (e.g., consumer-resource models).

Direct species-species interactions are characterized as positive, neutral, or negative (for either partner), resulting in six interaction archetypes (Großkopf and Soyer 2014; Amor and Bello 2019). The interactions are typically assumed to be pairwise (although higher-order interactions can be considered; Bairey et al. 2016; Grilli et al. 2017; Ludington 2022), constant over time, and independent of environmental conditions. Originally developed for macroscopic ecological communities, these simplifying assumptions enable analytical predictions about various aspects of the communities, such as the stability of the community (May 1972), the number of surviving species in unstable communities (Serván et al. 2018), or the prevalence of multiple stable states (Bunin 2017).

It is very tempting to adopt pairwise interaction models to describe microbial communities. Indeed, Lotka-Volterra models have been used successfully to predict aspects of microbial community dynamics and responses to environmental change in complex communities (Abreu et al. 2019; Lax et al. 2020; Hu et al. 2022). For instance, recent work has suggested that, regardless of the species or environmental conditions, higher temperatures favor slower-growing species (Lax et al. 2020; Abreu et al. 2022), or that the average interaction strength can alter the nature of the community dynamics in a predictable way (stable vs. chaotic) (Ratzke et al. 2020; Hu et al. 2022). These studies highlight that deliberately ignoring biological details can pave the way towards discovering principles of microbial ecology.

However, there are downsides to using Lotka-Volterra models for microbial communities. While pairwise interactions are experimentally relatively straight-forward to measure (Venturelli et al. 2018; Weiss et al. 2022), it remains difficult to do so for a large set of species. High-throughput methods have been developed to probe interactions of many bacteria across many environments (Kehe et al. 2019, 2021), but those methods are still far from mainstream and currently limited to genetically tractable focal organisms. Consequently, the relative prevalence and importance of different types of interactions (positive vs. negative) remains a topic of debate (Foster and Bell 2012; Ghoul and Mitri 2016; Kehe et al. 2021; Palmer and Foster 2022). Ultimately, measurements of pairwise interactions have limited predictive power for the competition outcomes in more complex communities (Friedman et al. 2017), and constant pairwise interactions are insufficient in quantitative predicting important features about communities, such as the diversity in different environmental conditions (Momeni et al. 2017; Mancuso et al. 2021). Lotka-Volterra models also fail to predict certain fundamental aspects of microbial communities, such as how community richness depends on the frequency of nutrient inputs (Mancuso et al. 2021) or the diversity of resources (Dal Bello et al. 2021).

As an alternative view to direct (pairwise) interactions, consumer-resource models explicitly consider interactions mediated by the excretion and exchange of chemical compounds with the environment. Mechanisms range from direct exchange of energy-rich compounds such as overflow metabolites (e.g., acetate) to electrons and protons changing the pH to antimicrobial compounds or non-metabolic public goods such as siderophores (Duan et al. 2009). Interactions based on chemical exchange between organisms also explain why spatial structure has such a big influence on microbial community composition and functions: diffusion and direct cell-to-cell exchanges limit interactions to small length scales, effectively decoupling cells at larger distances and making close spatial associations necessary (Mueller et al. 2013; Co et al. 2019, 2020; Dal Co et al. 2023). By combining metabolomics with microscopy, the exchange of metabolites between spatially associated microbes can also be visualized directly (Geier et al. 2020).

To parameterize consumer-resource models, information is needed about which substrates each species can consume, and which compounds they excrete. Growth characterization experiments provide data on substrate consumption, while metabolomics can measure changes in abundance of different chemical compounds in microbial cultures (Kosina et al. 2018; Blasche et al. 2021; Yu et al. 2022). Combining these data with mathematical models, mechanistic insight into microbial interactions can be gleaned (Blasche et al. 2021; Amarnath et al. 2023; Pontrelli et al. 2022). In many cases, the exchanged chemicals are metabolic intermediates that can be used by some community members as carbon, nitrogen, or energy sources (Gralka et al. 2020). Since so-called trophic interactions are directly related to metabolic processes in the individual community members, they can be understood from principles of microbial metabolism and physiology. For example, species with complementary metabolic strategies seem to be more likely to engage in interactions that are beneficial for at least one species than species that are metabolically similar (Giri et al. 2021; Kehe et al. 2021), presumably because the latter are likely to compete for the same resources. However, these results come from experiments of either limited taxonomic range or crossfeeding mechanisms. More research is needed to be able to predict trophic interactions based on species traits.

Box 1: **Mechanisms leading to the potential establishment of trophic interactions**

**Overflow metabolism**. Biochemical constraints can make shorter, but inefficient pathway the optimal choice for fast growth, leading to so-called overflow metabolism (Basan et al. 2015). A typical example of this is the fermentation of glucose by *E. coli* to acetate, which is excreted and can support the growth of other bacteria.

**Stress-induced excretions**. Osmotic stress, pH stress, and other forms of cellular stress can lead to metabolic imbalances. Metabolism may stall at some reaction, leading to accumulation and eventually excretion of the up-stream metabolites (Taylor et al. 2022), which can effect intricate crossfeeding dynamics in pairwise cocultures (Amarnath et al. 2023).

**Nutrient imbalances**. Limitation of one essential nutrient, e.g., nitrogen, may lead to the excretion of surplus carbon-rich compounds and vice versa (Pacheco et al. 2019). This is particularly evident in phytoplankton, where surplus carbon from photosynthesis is excreted in the form of soluble and insoluble polysaccharides, particular during N or P limitation (Myklestad 1995).

**Noise-averaging cooperation**. Recent theory predicts that noisy gene regulation (especially in bacteria) can lead to imbalanced enzyme and thus metabolite concentrations in individual cells (Lopez and Wingreen 2022). Sharing metabolite extracellularly averages out the individual imbalances, increasing growth rate at the population level. Excretions could be accomplished by dedicated or promiscuous transporters or passive membrane crossing of non-polar metabolites.

**Division of labor**. Some chemical transformations in multistep pathways may be carried out more efficiently by splitting the pathway between organisms, where each organism pays only part of the cost but also reaps only part of the benefit of running the pathway, a process called division of labor (Tsoi et al. 2018; Gowda et al. 2022). Intermediate products of the pathway are excreted by one organism and can be used as an energy or carbon sources by another. Division of labor may arise, e.g., when pathway intermediates are toxic (Goldschmidt et al. 2018) or when the intracellular concentration of enzymes in a cell is constrained (Thommes et al. 2019).

**Extracellular degradation**. Some functions, such as polysaccharide degradation, require an arsenal of extracellular enzymes produced by specialized bacteria (“degraders”). The action of these enzymes leads to release of soluble oligosaccharides that can be exploited by non-enzyme producers (“exploiters”) (Pollak et al. 2021). Both degraders and exploiter may excrete metabolites that benefit other community members (“crossfeeders”) for various reasons, including those given above (Pontrelli et al. 2022).

Understanding the physiological reasons leading to the potentially wasteful excretion of energy-rich metabolites is an important step in unraveling trophic interactions McKinlay 2023. Box 1 describes some of the mechanisms facilitating trophic interactions and studies where a deep exploration of the physiology of species in a community have led to the discovery of potential principles of microbial ecology. Given the various mechanisms giving rise to potential trophic interactions, it is no surprise that models that consider interactions through the uptake and excretion of (harmful or helpful) chemicals into the environment have recently enjoyed much success (Marsland et al. 2019; Niehaus et al. 2019; Pacciani-Mori et al. 2020; Amarnath et al. 2023). Dynamic models describing the growth of microbes and exchange of chemicals have been developed for individual species pairs (Momeni et al. 2017; Niehaus et al. 2019; Amarnath et al. 2023; Bloxham et al. 2022), and simplified versions of these models are straightforward to extend to complex communities (Goldford et al. 2018; Marsland et al. 2019). Combining high-quality genome-scale metabolic models (GEMs) for individual species into community models (Gottstein et al. 2016) allows for detailed predictions of the exchanged metabolites and the consequences of small changes to each species’ metabolic networks (e.g., gene inactivation). Community GEMs are very useful for the rational de-novo design of small consortia with a defined purpose, e.g., communities of lactic acid bacteria in the food industry. However, community GEMs require the input of curated GEMs for the individual species, which are often unavailable, and they must be experimentally validated.

Despite the consensus on the importance of species interactions for microbial communities, making quantitative predictions of complex communities remains challenging, particularly for environmental communities, where there is typically no information on physiological parameters or interactions between community members. Even when model and experiment agree, it can be a priori unclear which model will successfully reproduce the data, limiting our ability to make predictions for a new experimental system (Mancuso et al. 2021; Van Den Berg et al. 2022). Furthermore, mounting evidence suggests that the detailed dynamics of each of the thousands of bacterial species inhabiting a typical microbiome may be fundamentally unpredictable under certain conditions (Pagaling et al. 2014; Louca et al. 2016; Louca and Doebeli 2017; Pagaling et al. 2017; Ratzke et al. 2020; Estrela et al. 2022). Possible causes include a large number of alternative stable states, wherein stochasticity and historical contingency during community assembly (Zhou and Ning 2017) can steer communities towards different compositions from the micrometer scale (Szabo et al. 2022) to the ecosystem level (Bittleston et al. 2020; Vincent et al. 2023); strongly fluctuating or truly chaotic dynamics (Ratzke et al. 2020; Hu et al. 2022); and dominant higher-order interactions, which may lead to emergent species equilibria that are exceedingly difficult to disentangle beyond three or four species interactions (Bairey et al. 2016; Mickalide and Kuehn 2019; Sanchez-Gorostiaga et al. 2019). At present, therefore, there is no consistent bottom-up model of microbial communities, and the enormous complexity of wild microbial communities makes it appear unlikely that a single mathematical model can accurately describe the individual dynamics of all community members in a realistic environment.

### Top–down insight into communities

An alternative to the bottom-up approach discussed above is the top–down approach, which focuses on studying complex communities and their compositional and functional changes in response to their environment. A key question in this context is the degree to which the lower levels of organization (cells, species, and interactions) can predict community composition or function and the extent to which those community properties are emergent, representing true properties of the community. For emergent community properties, phenomenological models may be all that is practically relevant. In his 1972 essay, P. W. Anderson writes that “more is different” (Anderson 1972), referring to the emergence of new properties as a system becomes more complex. An example is the ideal gas law, which accurately describes the behavior of gases without considering the individual dynamics of gas molecules. The lesson here is that sometimes, simple models like the ideal gas law can effectively describe complex systems, even when an understanding of the individual components is lacking. The question arises whether similar principles could hold for microbial communities, where simple rules may emerge once a certain level of complexity is reached (Cui et al. 2021).

Recent studies have suggested such emergent simplicity in microbial communities. This simplicity is often described in terms of a coarse-grained description of the communities in terms of a small number of emergent variables. These variables can include statistical properties of species abundances, the abundance of certain functional groups, diversity changes in response to a change in environment, and statistical predictability of community functions. In the following, we will review some recent results derived from analyses of existing datasets as well as laboratory experiments, and extract potential principles that emerge.

At a macroecological level, statistical analyses of microbial community compositions across a large number of different ecosystems have revealed laws that shed light on the fundamental forces structuring communities (Grilli 2020). From this perspective, it appears that environmental fluctuations and self-limiting growth are dominant factors in shaping communities. Interactions between species are inferred to be relatively weak and sparse, as many statistical patterns in microbiome abundances can be reproduced without including species interactions. However, consumer-resource models in constant environments parameterized with appropriately structured random matrices can also reproduce many statistical properties of microbial communities, such as nestedness (Marsland et al. 2020).

Environmental sampling of comparable microbial ecosystems has demonstrated that physically and chemically similar ecosystems often have similar functional profiles, i.e., the relative abundance of functions like photosynthesis, sulfate reduction, etc., whereas species compositions may differ widely (Louca et al. 2016, 2017). This is because species in communities often overlap in terms of at least some of their functional traits (Mcgill et al. 2006; Litchman and Klausmeier 2008; Martiny et al. 2015), i.e., they are functionally redundant to varying degrees (Louca et al. 2018). Many functions are performed by a huge diversity of organisms (e.g., carbohydrate fermentation), but some functions may be highly phylogenetically conserved or even monophyletic (e.g., complete ammonia oxidation; Daims et al. 2015); in those cases, taxonomy and function become congruent.

Assigning traits to taxa can be a significant challenge, depending on the trait, its phylogenetic conservation, and the degree of understanding of the pathways involved. Identifying functional groups is therefore a major goal in microbial ecology. A recently developed algorithm addresses this difficulty by aiming to detect functional groups sequencing survey data groups solely from relative abundance data without taxonomic input and without explicit functional annotation (Shan et al. 2023). Such algorithms may help identify species or groups of species that produce specific compounds of interest or facilitate macroscopic outcomes in host-associated microbiomes.

A trait-based ecological approach to microbial communities can provide a new perspective on experimentally measured interactions networks by focusing on the interactions between functional groups rather than species-species interactions. This approach may be more appropriate for predicting community response to environmental changes. For example, consider the trophic interactions in polysaccharide-degrading communities between degraders, exploiters, and crossfeeders (Pollak et al. 2021) (described in Box 1 and reviewed in Sichert and Cordero 2021), which are characterized by a hierarchical structure: organic carbon flows from degraders to exploiters, and degraders and exploiters to crossfeeders, with little carbon flow in the reverse direction (Pontrelli et al. 2022). In these communities, different degraders appear to select for downstream community members, but not vice versa (Enke et al. 2019). For such a community, each represented by potentially many different species, do environmental perturbations have different effects depending on which trophic level is most impacted by the perturbation? The hierarchical structure suggests that perturbations targeting high trophic levels (e.g., degraders) have a relatively stronger community-level impact, but crossfeeders can also affect the hydrolytic activity of the degraders (Daniels et al. 2022). Similarly, crossfeeders in glucose-enriched soil communities selected for different degraders and not the other way around, as would be expected from trophic interactions alone (Estrela et al. 2022). More research is required to elucidate to what degree trophic interactions lead to hierarchical interaction networks.

In addition to inferring principles from large environmental datasets, systematic experimental efforts have quantitatively explored the changes of microbial communities with their environment. Examples include community changes with temperature (Lax et al. 2020; Abreu et al. 2023), mortality (Abreu et al. 2019), number of carbon sources (Dal Bello et al. 2021), carbon concentration (Gralka et al. 2022), type of carbon source (Estrela et al. 2021), and the strength of interactions (Ratzke et al. 2020). Enrichments of natural communities are often used for this type of experiment (Estrela et al. 2021), but complex synthetic communities are also a valuable tool (Vorholt et al. 2017; Kong et al. 2018). Synthetic communities offer exact control over the number and identity of species in a community and detailed (functional, physiological, etc.) characterization of the community members. By systematically manipulating the number of species in synthetic communities, theoretical predictions regarding community stability in different environments can be tested directly (May 1972; Fetzer et al. 2015; Hu et al. 2022). Synthetic communities of varying initial richness also enable the inference of structure–function landscapes, allowing the prediction of community function based solely on species presence/absence data, without explicit knowledge of dynamics or interactions (Gopalakrishnappa et al. 2022; Skwara et al. 2023).

### Tying it all together: the marine carbon cycle as an example

**Fig. 1:**
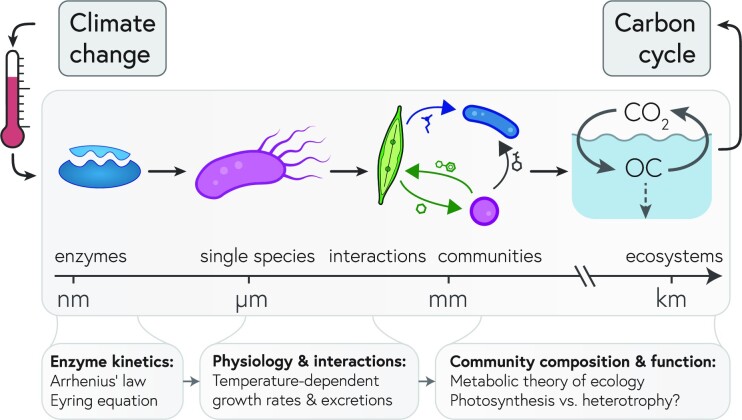
The effects of rising water temperature on the marine carbon cycle, through the lens of microbial communities, from enzymes to ocean-scale microbial activity. Temperature fundamentally tunes enzymatic activity, and these effects cascade up through various length scales, from the physiology of single species to species interactions to complex communities to ecosystem-level processes like biogeochemical cycles (OC = organic carbon). Eventually, the goal is to predict ecosystem-level responses to climate change, such as how increasing temperatures will shift the balance between photosynthetic carbon fixation and storage in the ocean relative to heterotrophic degradation of complex organic matter and subsequent release of CO_2_. While very simplified models, such as the metabolic theory of ecology, can serve as null models, a more detailed understanding is required to predict how temperature effects traverse scales from enzymatic rates to ecosystem functions.

Throughout this paper, we have primarily focused on general principles applicable across ecosystems. Here, as a concrete example, we will illustrate a scale traversal, from enzymes to entire ecosystem functions, by examining the marine carbon cycle and its response to increasing sea water temperatures caused by climate change (Fig. 1).

The ocean is a dynamic ecosystem whose microscopic inhabitants, i.e., phytoplankton and heterotrophic bacteria, cycle ∼50% of all carbon of our biosphere (Field et al. 1998). The cycling of carbon by marine microbes is driven by the collective metabolism of their communities, from carbon fixation and partial excretion of organic carbon by phytoplankton to the bacterial degradation of organic matter and exchange of metabolic by-products (Amin et al. 2012; Moran 2015; Gralka et al. 2020). Even small changes to these microbes and their communities can have a major impact on the planet (Cavicchioli et al. 2019), and marine microbial communities are already strongly affected by climate change (Behrenfeld et al. 2006; Brierley and Kingsford 2009). In this example, we will specifically focus on the increase of rising sea surface temperatures.

Increasing sea surface temperatures impact not only physicochemical processes in the ocean, such as the acidification, deoxygenation, and the degree of seasonal stratification (Keeling et al. 2010; Joint et al. 2011; Li et al. 2020), but also biological processes at all levels of organization (Arroyo et al. 2022). However, most studies focus exclusively on one level of organization, such as the physiological response of individual species or whole communities *in situ*. These approaches fails to reveal underlying principles because they do not connect traits of individual microbial species to the function of the whole microbiome. Consequently, current experiments and models are unable to provide accurate predictions for how climate change impacts carbon cycling by the marine microbiome (Taucher and Oschlies 2011).

At the molecular level, temperature influences enzymatic rates, with increasing rates below an optimal temperature described by Arrhenius’ law (Arroyo et al. 2022). The collective temperature sensitivities of the enzymes involved in microbial metabolism, including photosynthesis, central carbon metabolism, and extracellular carbohydrate-active enzymes, give rise to the temperature sensitivity of microbial activity. Despite the underlying complexity, the temperature sensitivity of microbial growth rate itself is also well described by Arrhenius’ law. Hence, the principles of enzyme kinetics seem to translate to cells, the next level of organization (Arroyo et al. 2022). Temperature sensitivities of maximal growth rates are available for many species (Smith et al. 2021), enabling realistic parameterization of mathematical models. However, carbon and nutrient availability modulate temperature sensitivities in ways that are not well understood (Pomeroy and Wiebe 2001; Hall and Cotner 2007; Thomas et al. 2017).

Moving from microbial growth to interactions, temperature can also impact metabolic processes that impact the excretion of metabolites, the production of antimicrobial compounds, and other factors. Thus, it is unclear how interactions between cells are expected to change with temperature. For example, photosynthesis rates generally increase with temperature (Kremer et al. 2017), leading to higher excreted carbon fluxes, which, in turn, facilitate more heterotrophic growth. Will increasing water temperature increase or decrease the net carbon flux into the ocean (fixation minus degradation)? So far, we are missing well-controlled experimental studies and models of these interactions to test existing predictions (Regaudie-De-Gioux and Duarte 2012). However, exact knowledge of microbial interactions may not always be required, and emergent principles at the level of interactions may be found that inform predictions about ecosystem changes with temperature. For instance, increasing temperature generically favors the slower grower in laboratory cocultures (Lax et al. 2020).

Emergent principles may allow us to move up through levels of organization, from interactions to communities. In the case of marine microbes, there is evidence that increasing temperatures favor slower growers even in complex communities on the ecosystem scale (Abreu et al. 2023). Other studies directly examine the functional effects of temperature changes on complex communities. Large-scale surveys have identified a remarkably simple relationship between certain ecosystem properties and temperature, which can be well described by Arrhenius’ law (Gillooly et al. 2001; Allen et al. 2002). This Metabolic Theory of Ecology (Gillooly et al. 2001; Brown et al. 2004) (MTE) ignores interactions between community members, which have been shown to modulate the temperature sensitivity of communities (García et al. 2023). Therefore, it is perhaps not a surprise that the predictions of the MTE at the microbial ecosystem level are not always borne out. For instance, the MTE predicts that diversity increases with temperature, but overall microbial diversity peaks ∼18°C (Thompson et al. 2017), far below the typical optimal growth temperature of most microbes. In the ocean, microbial diversity is maximal at extreme latitudes in the winter, i.e., when the surface temperature is lowest, in direct contradiction to the MTE (Ladau et al. 2013). Therefore, while the MTE offers a simple framework, it is insufficient to accurate model the temperature response of the ocean microbiome. Further research is needed to elucidate principles at the ecosystem level that will help develop improved models for predicting the consequences of climate change on the marine microbiome.

## Conclusion

The field of microbial ecology is at a turning point: 16S amplicon and metagenomic sequencing have delivered tremendous insight into the composition of microbial communities, and other meta-omics technologies continue to provide ever higher resolution views into the biological processes occurring in various microbial ecosystems. What is required next is a close collaboration between experimentalists and theorists and an integration of bottom-up and top–down approaches: to combine observational ‘omics data with quantitative experimental studies of individual community members and functional descriptions of whole communities to develop predictive models of microbial communities (Widder et al. 2016; Van Den Berg et al. 2022). High-throughput cultivation techniques, such as microfluidic (e.g., the K-Chip) (Kehe et al. 2019), millifluidic (e.g., the MilliDrop machine) (Boitard et al. 2015), or microwell (e.g., the bioMe plate (Jo et al. 2023), the iChip (Berdy et al. 2017)) approaches, deliver datasets with sufficient statistical power to derive principles of physiology and interactions across a wide range of non-model species. Bioreactor setups, increasingly available both commercially or as open-source projects, e.g., the UNLOCK platform (https://m-unlock.nl), evolvR (Wong et al. 2018), chi.bio (https://chi.bio), and piolab (https://pioreactor.com), allow for detailed functional measurements at high temporal resolution across a wide range of experimental conditions. Such experimental setups are key for providing the necessary data for testing mathematical models, which will both improve interpretation of existing omics data and enable quantitative predictions for and ration design of microbial communities.

## Data Availability

No datasets were generated or analysed during the current study.

## Notes

From the symposium “Large-scale biological phenomena arising from small-scale biophysical processes” presented at the annual meeting of the Society for Integrative and Comparative Biology virtual annual meeting, January 16–March 31, 2023.
